# Frequency comb transferred by surface plasmon resonance

**DOI:** 10.1038/ncomms10685

**Published:** 2016-02-22

**Authors:** Xiao Tao Geng, Byung Jae Chun, Ji Hoon Seo, Kwanyong Seo, Hana Yoon, Dong-Eon Kim, Young-Jin Kim, Seungchul Kim

**Affiliations:** 1 Max Planck Center for Attosecond Science, Max Planck POSTECH/KOREA Res. Initiative, Pohang, Gyeongbuk 376-73, South Korea; 2 Department of Physics, Center for Attosecond Science and Technology (CASTECH), POSTECH, Pohang, Gyeongbuk 376-73, South Korea; 3 School of Mechanical and Aerospace Engineering, Nanyang Technological University (NTU), 50 Nanyang Avenue, Singapore 639798, Singapore; 4 Department of Energy Engineering, Ulsan National Institute of Science and Technology (UNIST), Ulsan 689-798, South Korea; 5 Energy Storage Department, Korea Institute of Energy Research (KIER), Daejeon 305-343, South Korea

## Abstract

Frequency combs, millions of narrow-linewidth optical modes referenced to an atomic clock, have shown remarkable potential in time/frequency metrology, atomic/molecular spectroscopy and precision LIDARs. Applications have extended to coherent nonlinear Raman spectroscopy of molecules and quantum metrology for entangled atomic qubits. Frequency combs will create novel possibilities in nano-photonics and plasmonics; however, its interrelation with surface plasmons is unexplored despite the important role that plasmonics plays in nonlinear spectroscopy and quantum optics through the manipulation of light on a subwavelength scale. Here, we demonstrate that a frequency comb can be transformed to a plasmonic comb in plasmonic nanostructures and reverted to the original frequency comb without noticeable degradation of <6.51 × 10^−19^ in absolute position, 2.92 × 10^−19^ in stability and 1 Hz in linewidth. The results indicate that the superior performance of a well-defined frequency comb can be applied to nanoplasmonic spectroscopy, quantum metrology and subwavelength photonic circuits.

## Abstract (web summary)

Combining frequency combs and plasmonics promises highly precise timing and frequency standards in nanoscale devices. Here, the authors experimentally transfer a frequency comb to surface plasmons and then return it to its original comb form with little degradation.

## Graphical abstract

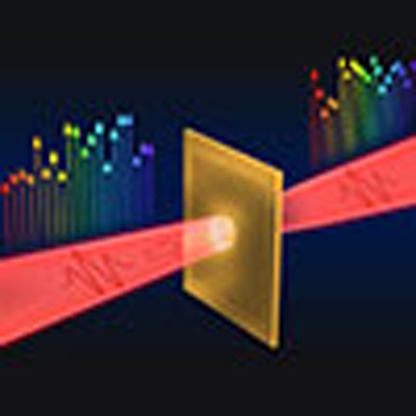

The frequency comb of mode-locked femtosecond lasers has led to remarkable advances in high-resolution spectroscopy^1^,^2^, broadband calibration of astronomical spectrographs^3^,^4^, time/frequency transfer over long distances^5^,^6^, absolute laser ranging^7^,^8^,^9^,^10^ and inter-comparison of atomic clocks^11^,^12^. It provides millions of well-defined optical modes over a broad spectral bandwidth with high-level phase coherence referenced to an atomic clock. Recently, the potential of frequency comb has expanded to microscopic applications; high inter-mode coherence within a short pulse duration enabled manipulating atomic qubits^13^, operating quantum logic gates and performing high-speed molecular detection by coherent Raman spectroscopy through harnessing inter-mode beat frequencies between two frequency combs at different repetition rates^14^.

Coupling surface plasmons (SPs)^15^,^16^, collective charge oscillations produced by the resonant interaction of light and free electrons on the interface of metallic and dielectric materials, to frequency comb creates numerous advantages. First, SP can allow for the frequency comb to access nanoscopic volumes that surpass the diffraction limit^17^. Second, the field enhancement by localized SP enables the highly sensitive detection of weak signals, even from a single molecule (for example, surface-enhanced Raman scattering)^18^. Third, next-generation photonic devices and circuits can be implemented within a small subwavelength volume by all-optical control of light properties (amplitude, phase and polarization state) in plasmonic nanostructures within ultrafast time scales^19^,^20^,^21^,^22^. However, the superior performance of the frequency comb, such as absolute frequency uncertainty, high-frequency stability and narrow linewidth, could deteriorate during the photon-plasmon conversion process. For exploring novel combination of frequency comb and SP resonance, it is prerequisite to verify that frequency comb maintains its performance under plasmonic resonance; however, there have been no studies to date.

In the following, we report that frequency comb successfully maintains core performances in photon-plasmon conversion by exploiting plasmonic extraordinary transmission through a subwavelength plasmonic hole array. This implies that the original frequency comb can be transformed into a form of plasmonic comb on metallic nanostructures and reverted to an original frequency comb without noticeable degradation in absolute frequency position, stability and linewidth. The superior performance of well-defined frequency combs can therefore be applied to various nanoplasmonic spectroscopy, coherent quantum metrology and subwavelength photonic circuits.

## Results

### Frequency comb transferred by SP resonance

**Figure 1:**
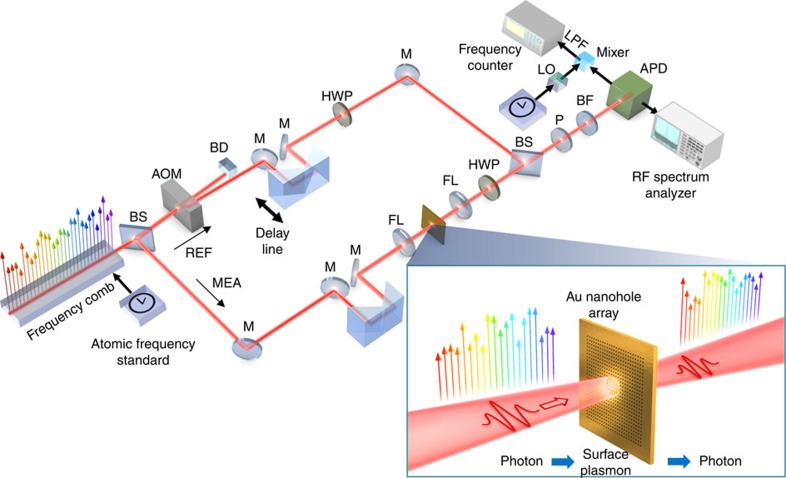
Generation and characterization of plasmonic frequency comb. Part of the frequency comb experiences plasmonic mode conversion by passing through the plasmonic sample. The sample consists of a subwavelength nanohole array on an Au thin-film, enabling the conversion from photon to SP (from SP to photon). The other part of the frequency comb is used as a reference beam to compare with the frequency comb passed through the plasmonic sample. The frequency combs at two different paths are combined and monitored by APD. The characteristics of the frequency comb at measurement path are analysed by an RF spectrum analyser and a frequency counter. APD, avalanche photo-detector; BD, beam dumper; BF, band-pass filter; BS, beam splitter; FL, focusing lens; HWP, half-wave plate; LO, local oscillator; LPF, low-pass filter; M, mirror; MEA, measurement beam path; P, polarizer; REF, reference beam path.

Figure 1 shows the experimental apparatus to characterize the conservation of frequency comb for the conversion from photon to SP. The frequency comb is split into reference and measurement beams; one part of the beam transmits through an acousto-optic modulator (AOM) for a frequency shift of 40 MHz to construct a reference frequency comb and the other part of the beam passes through the plasmonic sample. The frequency comb structure in SP resonance was generated by the exploitation of a metallic nanohole array used for extraordinary optical transmission (EOT) that converted photon into SP. The small diameter of each hole prevents light passing through the sample based on classical optics. However, the SP-mediated tunnelling effect of nanohole array drastically enhances optical transmittance^23^. These intriguing optical phenomena have been studied widely for high-resolution chemical sensing, ultrafast optical modulation, wavelength-tunable optical filtering and subwavelength lithography^24^,^25^. The physical origin of EOT has been attributed to resonant SP polaritons (SPPs)^26^. The appropriate geometrical and material parameters of nanohole array excite the SPP mode that allows the transmission of light that contains plasmonic information inside an EOT sample. The resonant nature of the SP changes the transmitted spectral distribution, depending on sample design, input polarization and incident angle. Plasmonic EOT can also induce wavelength-dependent changes in optical frequency and phase in addition to wavelength-dependent transmittance. The optical frequency of a single frequency comb mode transmitted through the plasmonic sample via SP resonance (*f*_MEA_) can be expressed as


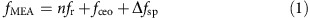


where *f*_r_ is the pulse repetition frequency*, f*_ceo_ the carrier-envelope offset frequency, and Δ*f*_sp_ the frequency and phase change generated by SP resonance. Meanwhile, the optical frequency of the single mode passing through the reference path (*f*_REF_) can be expressed as


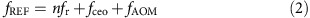


where *f*_AOM_ denotes the intentional frequency shift by AOM. The detection of the heterodyne beat-frequency generated by the interference between the reference and measurement beams enables the measurement of optical frequency difference, (*f*_REF_−*f*_MEA_) at a radio-frequency (RF) regime using a fast avalanche photodiode. This resultant frequency difference can be simplified to *f*_AOM_−Δ*f*_sp_, where *f*_AOM_ works as the high-frequency carrier to isolate Δ*f*_sp_ from the relatively strong low frequency noise components.

### Plasmonic extraordinary transmission

**Figure 2:**
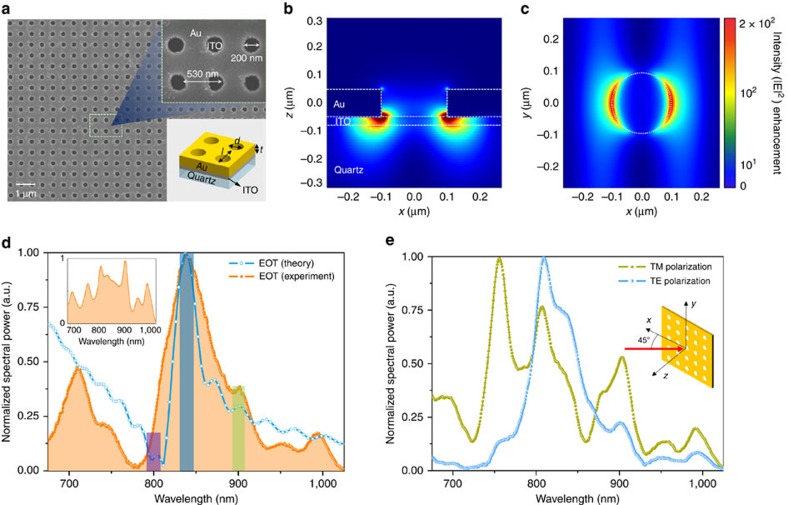
Numerical simulation and characterization of fabricated plasmonic sample. (**a**) Scanning electron microscope image of the fabricated subwavelength nanohole array for plasmonic EOT. The fabricated nanohole has the diameter (*d*) of 200 nm, pitch (*l*) of 530 nm and thickness (*t*) of 100 nm on 25-nm-thick ITO-coated quartz substrate. (**b**) Calculated intensity distribution of an plasmonic sample taken from the side. (**c**) Calculated intensity distribution at the interface between Au and ITO layer. (**d**) Theoretical (blue line) and experimental (orange line) spectrum of transmitted frequency combs through the plasmonic sample. Purple, blue and green bars represent the selected spectral components (800, 840 and 900 nm) to characterize frequency comb, respectively. Inset (top left) shows the original spectrum of the frequency comb. (**e**) Polarization dependent transmission spectrum through the plasmonic sample at an incident angle of 45°.

For transmitting frequency combs through the subwavelength holes by SP resonance, there are three important geometric parameters: hole diameter (*d*), hole pitch (*l*) and Au film thickness (*t*; Fig. 2a). For maximum optical transmission at a wavelength of 840 nm, three parameters were optimized by solving Maxwell's equations using finite-difference time-domain (FDTD) method. Figure 2b,c show the calculated plasmonic field distribution through the optimized sample. The electric field around the hole was significantly enhanced by SP in the periodic apertures, delivering the optical energy through the hole. Figure 2a shows the scanning electron microscope image of the fabricated nanohole array; all dimensions were matched with optimized design parameters within a geometric error of <5%. Figure 2d shows that the transmitted optical spectrum coincided with the numerical FDTD results and validated the numerical analysis. Minor deviations between the two spectrums are expected by focusing geometry onto the plasmonic sample. The plasmonic resonance conditions are dissimilar in given transverse electric-transverse magnetic polarization if the angle of incidence is not surface normal. As a result, plasmonic sample shows different transmission spectra for transverse electric-transverse magnetic polarization at the incidence angle of 45° (Fig. 2e); therefore, optical transmission of our sample is dominated by the plasmonic EOT, not classical diffraction theory.

### Frequency comb structure after plasmonic transmission

**Figure 3:**
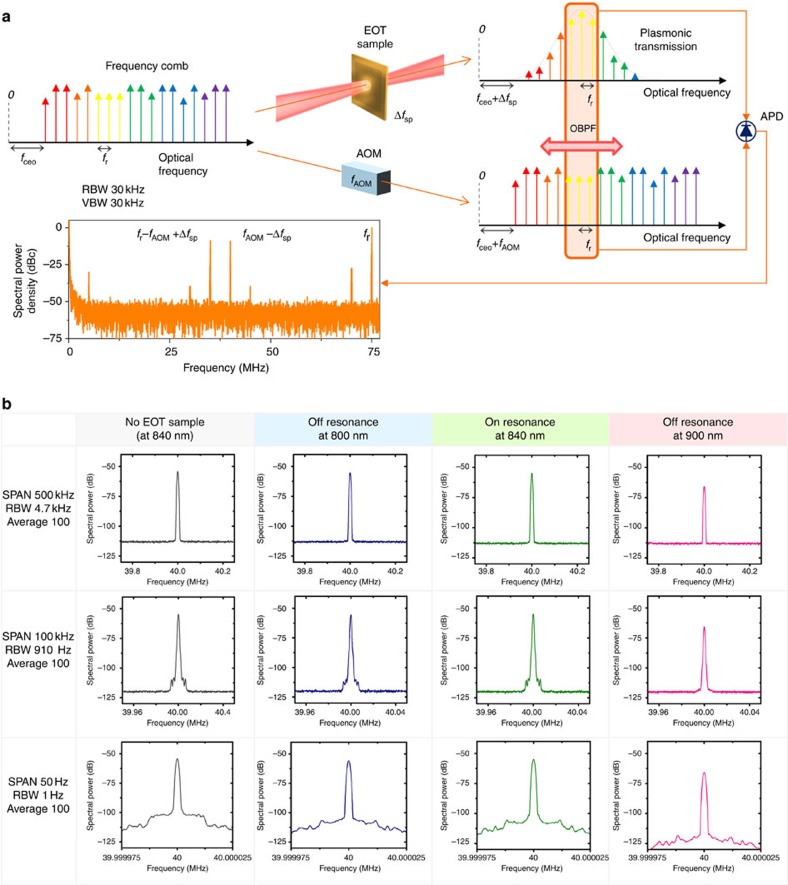
Evaluation of the plasmonic frequency comb by EOT. (**a**) Generation of RF beats by the interference between frequency-shifted (40 MHz) reference combs and plasmonic EOT combs. The beat spectra of plasmonically transmitted frequency comb and the reference comb are measured at three wavelengths: one at a strong plasmonic resonance position (a 840-nm centre wavelength with a 10-nm bandwidth), two at off-resonance positions (a 800-nm centre wavelength with a 40-nm bandwidth and 900 nm with a 10-nm bandwidth) using three optical band-pass filters. These are compared with a beat spectrum at a 840-nm wavelength, acquired without the plasmonic sample. (**b**) Linewidth measurement of RF beats with different span, RBWs and VBWs. There was no noticeable linewidth degradation by the plasmonic transduction (<1 Hz, limited by RBW of the instrument). APD, avalanche photo-detector; OBPF, optical band-pass filter; VBWs, video bandwidths.

The transmitted frequency combs through the plasmonic sample results in an interference with the reference frequency comb to verify the frequency comb structure after the photon-plasmon mode conversion by the EOT (Fig. 3a). For comparative analysis, interference signals were obtained at three different wavelength regimes with optical band-pass filters, representing on-resonance (840 nm) and off-resonance (800 and 900 nm) positions.

The coherence of a large number of frequency comb modes can be deteriorated by temporal and spectral plasmonic dispersion, phase noise and frequency noise during the propagation through the plasmonic EOT sample. The frequency comb fundamentally suffers from phase and frequency noises when passing through the optical medium (for example, ambient air and optical fibre) exposed to environmental variations, such as vibration, temperature variation and humidity change. Therefore, it has been an important task to monitor and compensate the temporal and spectral dispersion, phase noise and frequency noise generated in the medium, as reported through long optical-fibre^6^ and through ambient air^27^. SPs also suffer from the dispersion and phase change by the medium and environmental disturbances, which have not been investigated with the frequency comb for their quantitative or qualitative analysis. Propagating SPs through the EOT sample experience phase delay depending on their wavelengths and spatial locations before and after tunnelling through each subwavelength hole; this phase delay can be additionally induced by the plasmonic dynamic damping, imperfect sample geometry, surface roughness of the metal film or air refractive index change around the sample. Therefore, the total summation of the electromagnetic waves at the output side of each hole may contain temporal and spectral dispersion, phase distortion and frequency change.

Most noise sources of the frequency comb can be categorized into intra-cavity and extra-cavity sources; intra-cavity noise sources (including cavity length change, cavity loss fluctuations and pump noise) cause frequency noise whereas extra-cavity noise sources (induced by path-length fluctuation, shot noise from the limited power or noise generated during supercontinuum generation) result in time-varying phase noise floor^5^. In this investigation, plasmonic mode conversion by the EOT was considered as an extra-cavity noise source that provided wavelength-dependent power attenuation, phase shift and frequency noise, similar to the supercontinuum generation process. Noise contributions should be observed at *f*_AOM_−Δ*f*_SP_ in the form of linewidth broadening, frequency shift, signal-to-noise (S/N) ratio reduction, increased phase noise or a higher Allan deviation if the plasmonic frequency comb suffers from phase or frequency noise during the plasmonic mode conversion.

Linewidth broadening and S/N ratio reduction in plasmonic mode conversion process was initially evaluated by measuring RF beat linewidth of *f*_AOM_−Δ*f*_SP_ at three different wavelength regimes (Fig. 3b). With different resolution bandwidths (RBWs), there was no substantial degradation in the linewidth at 840 nm before and after the installation of the plasmonic sample in the beam path. The high-level S/N ratio of ∼60 dB beat signal indicates that the plasmonic EOT provide no significant phase noise to the frequency comb.

**Figure 4:**
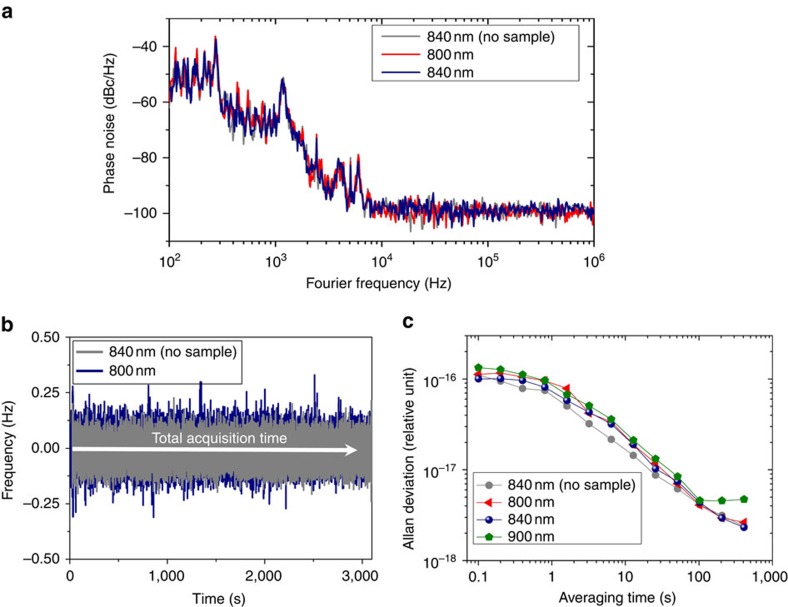
Plasmonic frequency comb: phase noise and frequency stability. (**a**) Phase noise spectra at a 1.2-GHz RF carrier at on- and off-resonance wavelengths. (**b**) Time trace of the beat frequency with and without the plasmonic sample over 3,000 s. (**c**) Allan deviations of frequency stability with varying average time at the positions of on-resonance and off-resonance wavelengths.

Phase noise and frequency stability was measured for the quantitative analysis of frequency-dependent noise contributions. Figure 4a shows the phase noise spectrum obtained by monitoring one of high harmonics of the beat frequencies at ∼1.2 GHz with and without the plasmonic sample; this confirms that there was no noticeable frequency noise inclusion. For high-precision frequency position measurement, the beat frequency between reference and measurement frequency comb was measured by a frequency counter for 3,000 s, resulting in 0.24 mHz frequency difference with a s.d. of 61 mHz (Fig. 4b). This corresponds to 6.51 × 10^−19^, which proves that plasmonic mode conversion provides no substantial degradation in the frequency accuracy of the frequency comb. The stability of the beat signal was measured to be 4.08 × 10^−18^ without the plasmonic sample, 4.37 × 10^−18^ with the plasmonic sample at resonance wavelength of 840 nm for an averaging time of 100 s, respectively (Fig. 4c). At the off-resonance wavelength, the stability of beat signal was 4.59 × 10^−18^, signifying almost no difference between on- and off-plasmonic resonance stabilities. All the experiments pointed that plasmonic mode conversion causes no substantial degradation to the frequency comb in terms of linewidth, frequency position, S/N ratio and frequency stability.

## Discussions

All hundreds of thousands optical modes in the frequency comb were firstly converted from photonic to plasmonic mode at the input side of the plasmonic EOT sample and then reverted to photonic mode at the other output side of the sample. It is known to be practically difficult to directly measure the optical frequency of the plasmonic mode so the characteristics of the plasmonic comb were measured here in the far field. Because the plasmonic and photonic modes are assumed to be mutually coherent, if there is any change in the frequency comb characteristics during the plasmonic propagation (in plasmonic mode) through the sample, it should be monitored at the output side in the far field (in photonic mode). Therefore, the beat-frequency detection using the transmitted photonic mode in the far-field regime enabled us to compare the qualities of the plasmonic comb with the original frequency comb, which cannot be implemented in the near-field regime. As the result of the comparison, there were no noticeable degradation in linewidth, frequency shift, S/N ratio, phase noise and Allan deviation. This implies that SP, the collective electrons, can be regarded as information carrier as precise as the optical frequency comb.

The frequency comb passing through the plasmonic EOT sample experiences the different physical process with the light reflection at a metallic mirror. Although both of the SP resonance and the surface reflection are governed by free-electron oscillation in conduction band of metals, the SP resonance additionally requires the specific momentum matching between incident photon and SP, whose relationship is determined by the plasmonic dispersion relation. Therefore, it is natural to maintain the coherence during the light reflection at metal surface (governed by frequency conservation), which is not the case in plasmonic structures (governed by frequency and momentum matching). Once the incident photon (in photonic mode) is converted into SP, it will propagate through the metal as the form of SPPs (in plasmonic mode). This plasmonic propagation causes temporal and spectral dispersions, phase variations and frequency changes, which may degrade the inherently high coherence of the optical frequency comb.

Plasmonic EOT is governed by not only the hole geometry^28^ but also hole pitch. Therefore, the incidence angle tuning of the input beam can provide the change in plasmonic coupling mode without dimensional changes, which can possibly cause some degradation in the frequency characteristics of the frequency comb by providing different plasmonic field distribution and enhancement. To test this, the beat spectrum was monitored while the sample was rotated by up to 45° (for transverse magnetic wave) as shown in Figure 2e. For the given condition, all frequency characteristics were maintained in the same level with normal incidence case, which shows that no performance degradation exist depending on plasmonic coupling or geometrical parameters of the sample.

The linewidth broadening by plasmonic EOT was evaluated to be <1 Hz, which is limited by RBW of the instrument in use (Fig. 3b). A single RF beat-frequency corresponds to the superposition of small RF beat contributions of >10^4^ frequency comb modes, which proves that there is no significant wavelength-dependent frequency or phase noise during the plasmonic EOT. There was minor increase in spectral power in the pedestal peaks at 12, 17 and 21 Hz when the frequency comb passed through the plasmonic sample; this is expected to be caused by the vibrational and thermal noises at the plasmonic sample. The beat frequency, *f*_AOM_−Δ*f*_SP_, was found to be exactly the same as the driving frequency of the AOM in all measured spectra shown in Fig. 3b, which implies that the absolute frequency position is well maintained in the plasmonic mode. The ambient temperature and vibration on EOT sample were not intentionally controlled so as to evaluate the performance in normal laboratory environment conditions. Our results show that the frequency comb structures are well maintained under environmental disturbances, for example, temperature variation, mechanical vibration and air fluctuation. This will enable us to develop high-sensitivity frequency-comb-referenced SP sensors working in harsh environments. The phase noise spectra in Fig. 4a also shows a number of minor peaks at 0.2, 1.5, 300 and 600 kHz other than the low-frequency spectral peaks at 12, 17 and 21 Hz observed in Fig. 3b. At higher frequency than 10 kHz, there is a flat noise floor without other spectral peaks or broad pedestals. In S/N ratio measurement, the S/N ratio theoretically could reach 68∼75 dB in a 100 kHz RBW because there are 10^4^∼10^5^ frequency comb modes in the pass-band of the optical filter transmittance. The experimental S/N ratio with the plasmonic sample on-resonance position was ∼60 dB; this minor deviation could come from imperfect intensity balancing, polarization matching and spatial beam mode-matching. The S/N ratio at 900 nm was 54 dB, relatively lower than that at 800 nm because the quantum efficiency of the avalanche photo-detector at 900 nm is ∼20% lower than that at 800 nm and the filter bandwidth at 900 nm is 25% of that at 800 nm.

In this article, we have studied SP resonance effects on frequency comb structure in the plasmonic EOT of light through a subwavelength metallic nanohole array. The frequency comb was transduced to plasmonic mode in the sample and reverted to photonic mode without significant changes in linewidth, frequency shift, S/N ratio, phase noise and Allan deviation. The linewidth broadening was <1 Hz (instrument limited), frequency inaccuracy was 6.51 × 10^−19^, S/N ratio was higher than 60 dB, Allan deviation increased by 2.92 × 10^−19^ at 100 s averaging time. This outstanding frequency comb performance in plasmonic nanostructures enables a highly sensitive, high accurate and broadband measurement with direct traceability to standards. This inclusion of frequency comb has the potential to accelerate progresses in various plasmonic applications such as bio-chemical spectroscopy or sensing, quantum optics and sub-diffraction-limit biomedical-imaging. With the aid of SP, frequency-comb-referenced high-speed coherent anti-stokes Raman spectroscopy^14^ can be implemented in much smaller nanoscopic volume being requested for single-molecule detection, for example, surface-enhanced coherent anti-stokes Raman spectroscopy^29^. A large number of optical modes in a frequency comb as the time and frequency standard can be coupled at the same time with SP for broadband quantum metrology for entangled atomic qubits or information carrier in subwavelength scale^13^,^30^. Localized field enhancement of SP will enable highly efficient nonlinear optics^31^ coupled with high precision of frequency comb, which is prerequisite for novel sub-diffraction-limit nonlinear biomedical imaging and spectroscopy.

## Methods

### Frequency comb

A Ti:sapphire femtosecond laser delivers 4.8 fs pulses at a repetition rate of 75 MHz over a broad spectral bandwidth from 1.03 to 2.06 eV (Venteon UB, Venteon). For establishing a frequency comb, the pulse repetition frequency (*f*_r_) and carrier-envelope offset frequency (*f*_ceo_) were precisely locked to a reference Rb atomic clock (FS725, Stanford Research Systems) with the aid of a *f*–2*f* interferometer and phase-locked control loops (AVR32, TEM-Messtechnik & XPS800-E, Menlosystems). One part of the beam was diverted to and transmitted through an AOM for the frequency shift of 40 MHz to construct a reference frequency comb. If the plasmonic frequency comb suffers from the phase or frequency noise during the plasmonic mode conversion, the noise contributions should be observed at *f*_AOM_−Δ*f*_SP_ in the forms of linewidth broadening, frequency shift, S/N ratio reduction, increased phase noise or higher Allan deviation. The frequency comb excited the plasmonic sample with the whole-broadband spectrum in a loose focusing geometry with an aspheric lens of 100 mm focal length. The focused peak intensity at the plasmonic sample was set to be <0.1 MW cm^−2^ not to exceed the thermal damage threshold (∼1 TW cm^−2^ for Au). Input polarization state was set linear and its direction is parallel to the *x* axis of periodic holes on plasmonic sample as denoted in Fig. 2e.

### Plasmonic EOT sample: design and development

We exploited FDTD solution (XFDTD8.3, Lumerical) to solve Maxwell's equation for plasmonic near-field distribution and transmitted spectrum. Through a series of iterative computations, the optimal geometric parameters were determined as *d*=200 nm, *l*=530 nm and *t*=100 nm. The thickness, *t* was designed to be much thicker than the Au skin depth (∼20 nm) here to block the direct transmission through the Au film. The designed nanohole array was fabricated using electron-beam lithography (Raith 150) onto 25-nm-thick ITO-coated quartz substrate.

### Evaluation of plasmonic frequency comb

For comparative analysis, interference signals were obtained at three different wavelength regimes – one at plasmonic on-resonance (840 nm) and the others at off-resonance positions (800 and 900 nm) – using optical band-pass filters. The resulting interference beat signal was obtained by high-speed avalanche photodiode and analysed using a high-resolution RF spectrum analyzer (N9020A, Agilent) and a RF frequency counter (53230A, Keysight Technologies). An exemplary RF spectrum is shown in Fig. 3b; the repetition rate (*f*_r_) is located at 75 MHz, the beat frequency (*f*_AOM_−Δ*f*_sp_) between the frequency-shifted reference frequency comb and the plasmonic frequency comb is at ∼40 MHz, and the beat frequency (*f*_r_−*f*_AOM_+Δ*f*_sp_) between the other nearby reference frequency comb modes and the plasmonic frequency comb is at ∼35 MHz. Other minor spurious peaks are due to the imperfect sinusoidal modulation of AOM; their positions match with beat frequencies between the *f*_AOM_-harmonics and the reference frequency comb of 2*f*_AOM_−*f*_r_, 2*f*_r_−3*f*_AOM_, 3*f*_AOM_−*f*_r_ and 2(*f*_r_−*f*_AOM_).

## Additional information

**How to cite this article:** Geng, X. T. *et al.* Frequency comb transferred by surface plasmon resonance. *Nat. Commun.* 7:10685 doi: 10.1038/ncomms10685 (2016).
